# Red ginseng extract enhances mitochondrial function and alleviates immunosenescence in T cells

**DOI:** 10.1016/j.jgr.2025.05.004

**Published:** 2025-05-20

**Authors:** Ho Yeop Lee, Jingwen Tian, Ha Thi Nga, Thi Linh Nguyen, Ji Sun Moon, Hyo Ju Jang, Alfin Mohammad Abdillah, Jieun Lee, Sang Hyeon Ju, Seung Ho Lee, Hun Kun Ko, Minho Shong, Hyon-Seung Yi

**Affiliations:** a Laboratory of Endocrinology and Immune System, Chungnam National University School of Medicine, Daejeon, Republic of Korea; b Department of Medical Science, Chungnam National University School of Medicine, Daejeon, Republic of Korea; c Department of Internal Medicine, Chungnam National University School of Medicine, Daejeon, Republic of Korea; d Graduate School of Medical Science and Engineering, Korea Advanced Institute of Science and Technology, Daejeon, Republic of Korea; e R&D Headquarters, Korea Ginseng Corp., Gyeonggi-do, Republic of Korea; f College of Nursing, Chungnam National University, Daejeon, Republic of Korea

**Keywords:** Red ginseng extract, Aging, Mitochondria, Immunosenescence, T cell

## Abstract

**Background:**

Mitochondrial function is essential for immune cell regulation, and its decline is linked to aging and chronic diseases. Impaired activity contributes to inflammation and reduced immunity. This study explores Red ginseng extract (RGE)'s potential in enhancing mitochondrial function and immune cell viability, offering benefits in mitigating immunosenescence.

**Methods:**

T cells and macrophages from young (12-week-old) and aged (20-month-old) mice were treated with RGE to assess mitochondrial function and cell viability. Flow cytometry evaluated immune cell populations and cytokine expression in splenocytes, while single cell transcriptomics analyzed RGE-induced transcriptional changes in T cells and macrophages.

**Results:**

RGE treatment improved mitochondrial oxygen consumption rate and glycolytic function in CD4^+^ and CD8^+^ T cells from both young and old mice, though effects were more pronounced in young cells. In aged mice, RGE administration resulted in higher proportions of naive T cells and reduced expression of senescence and exhaustion markers. Flow cytometry analysis indicated a decrease in pro-inflammatory cytokines IFN-γ and TNF-α in T cells, along with a reduction in IL-17-producing T cells. Single cell transcriptome analysis revealed downregulation of aging markers (*Cd28* and *Cd27*) and increased expression of mitochondrial complex genes, supporting RGE's role in enhancing mitochondrial function.

**Conclusion:**

RGE treatment enhances mitochondrial function and attenuates T cell senescence and exhaustion in aged immune cells, likely contributing to immune resilience against age-associated inflammation. This study highlights the potential of RGE as a therapeutic intervention for improving immune function and reducing the effects of immunosenescence, offering valuable insights into mitigating age-related immune decline.

## Graphical abstract

**Figure:**
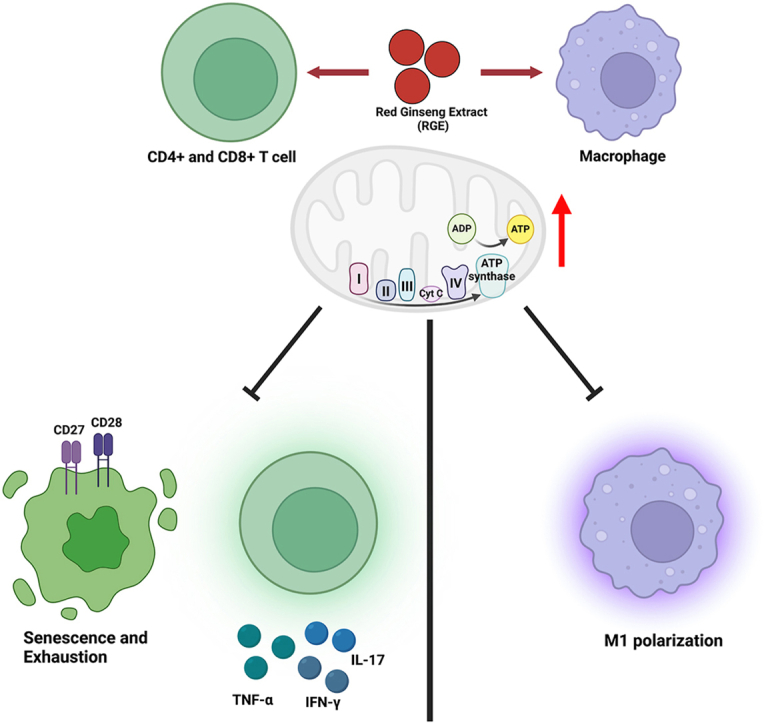

## 1 Introduction

Mitochondrial function plays a crucial role in regulating immune cell activity, and its dysregulation is closely linked to the aging process and chronic metabolic disease [[1], [2], [3]]. Aging is marked by a decline in mitochondrial efficiency within immune cells, such as T cells and macrophages, resulting in reduced respiratory capacity, decreased mitochondrial membrane potential, and increased production of reactive oxygen species [1,4]. Considering the relevance of mitochondria in T cells and macrophages, the disruption of mitochondrial function in these immune cells during aging has been associated with functional declines [4,5]. This dysfunction contributes to weakened immune responses, which are linked to age-associated heightened inflammation, and an increased susceptibility to infections and chronic metabolic diseases [4,6,7]. Therefore, exploring strategies to enhance mitochondrial function in immune cells is essential to counteract age-related immunosenescence.

Mitochondrial health is increasingly recognized as critical for sustaining immune function across the lifespan. Age-associated mitochondrial decline in immune cells leads to reduced cellular energy production, oxidative stress accumulation, and eventual immune cell exhaustion, collectively contributing to immunosenescence and greater morbidity and mortality in older adults. Despite increased focus on mitochondrial-targeted therapies, effective interventions for immune aging remain limited. Red ginseng extract (RGE), known for its antioxidant and anti-inflammatory properties, holds promise for preserving immune resilience through mitochondrial support. Investigating RGE's effects on aged immune cells thus represents a novel approach for improving healthspan and immune robustness.

As a traditional medicine, RGE has been widely used in East Asian countries, including China, Japan, and Korea, and continues to gain worldwide popularity for its medicinal properties [8]. Emerging evidence suggests that RGE may improve dysregulated immune cells-mediated inflammatory diseases. RGE can regulate Th17 and reciprocally promote Treg cells by inhibiting the phosphorylation of STAT3 [9]. Moreover, RGE attenuates atopic dermatitis-like skin lesions by suppressing Th2 immune response or inhibiting serum IgE and IL-6 release and chemokine expression in a murine model [10,11]. RGE-mediated increases in CD8^+^ T cells and CD11c + dendritic cells can be a mechanism contributing to desirable clinical outcomes of diminishing or preventing mouse body weight loss upon infection with RSV [12]. Given RGE's effects on inflammatory and infectious diseases, investigating its potential to improve mitochondrial function in immune cells is both timely and relevant. Research into RGE's potential to mitigate immunosenescence holds significant value.

In this study, we investigated the effect of RGE on mitochondrial oxidative phosphorylation and cell survival in T cells and macrophages isolated from young (12-week-old) and aged (20-month-old) mice. Furthermore, we assessed the immunomodulatory potential of oral administration of RGE in young and aged mice through flow cytometry and single cell transcriptome analysis of splenic immune cells.

## 2 Methods

### 2.1 Mice

C57BL/6 *Wild-type* mice were purchased from Jackson Laboratory, and were kept in a specific pathogen-free environment at the Preclinical Research Center of Chungnam National University Hospital. All mice were maintained in a controlled environment (12-h light/dark cycle; humidity, 50–60 %; ambient temperature, 22 ± 2 °C) and fed a normal chow diet (NCD), or NCD with RGE during 20 weeks. All animal experiments were approved by the Committee on the Ethics of Animal Experiments of Chungnam National University Graduate School of Medicine (CNUH-017-A0048, Daejeon, Korea) and were performed according to the institutional guidelines for the care and use of laboratory animals.

### 2.2 Preparation of chow diet with Korean Red Ginseng extract

In the present study, G1899 was prepared by the Korea Ginseng Corporation (Republic of Korea) as follows. KRG was prepared by steaming 6-year-old P. ginseng in compliance with the requirements of the International Organization for Standardization (ISO) 19,610:2017. Both P. ginseng and KRG complied with quality control criteria for acceptable levels of pesticides and contaminants. KRG was extracted seven times using 10 vol of distilled water at 85 °C for 10 h and concentrated under vacuum at 60 °C until it reached above 60° brix. Finally, the concentrated extract was dehydrated using a spray-drying method to remove moisture and obtain a light-brown powder (G1899). Table 1 shows the composition of G1899. Mice were fed a pelletized chow diet supplemented with G1899 [0.2 % (Low_200 mg/kg) and 0.4 % (High_400 mg/kg) G1899].

**Table 1:** Composition of major components of G1899 used in this experiment.

Components		G1899 (mg/powder g)
Ginsenosides	Rg1	1.41
	Re	1.57
	Rf	1.79
	Rh1	1.43
	Rg2s	1.85
	Rb1	7.72
	Rc	2.99
	Rb2	2.60
	Rd	0.99
	Rg3s	2.58
	Rg3r	1.31
	Total contents	26.24
AFG(Arginine-Fructose-Glucose)		19.63
AP(Acidic Polysaccharide)		85.77

### 2.3 Isolation of CD4^+^ and CD8^+^ T cells in mice

Spleens were harvested from mice and mechanically dissociated to obtain a single cell suspension. The spleen tissue was passed through a 70 μm cell strainer into cold phosphate-buffered saline (PBS) containing 0.5 % bovine serum albumin (BSA) and 2 mM EDTA (MACS buffer). The cell suspension was centrifuged at 300×*g* for 10 min at 4 °C, and the supernatant was discarded. The cell pellet was resuspended in 5 mL of red blood cell lysis buffer (BioLegend) and incubated for 2 min at room temperature to remove erythrocytes. The reaction was stopped by adding 10 mL of MACS buffer, followed by centrifugation at 300×*g* for 5 min at 4 °C. After washing, the cells were resuspended in MACS buffer at a concentration of 10^7^ cells per 80 μL. CD4^+^ T cells were isolated using the CD4^+^ and CD8^+^ MicroBead kit (Miltenyi Biotec) according to the manufacturer's instructions. Briefly, cells were incubated with CD4^+^ or CD8^+^ MicroBeads (20 μL per 10^7^ cells) for 15 min at 4 °C. Following incubation, cells were washed by adding 2 mL of MACS buffer per 10^7^ cells and centrifuged at 300×*g* for 10 min at 4 °C. The supernatant was carefully removed, and the cell pellet was resuspended in 500 μL of MACS buffer. The labeled cell suspension was applied to an MS or LS column (Miltenyi Biotec) placed in a magnetic field using a MACS separator. The column was washed three times with 500 μL of MACS buffer to remove unlabeled cells. CD4^+^ or CD8^+^ T cells were eluted by removing the column from the magnetic field and flushing it with 1 mL of MACS buffer. The purity of the isolated CD4^+^ or CD8^+^ T cells was assessed by flow cytometry using an anti-CD4 antibody (BioLegend). Isolated CD4^+^ or CD8^+^ T cells were then used for subsequent experimental assays.

### 2.4 Flow cytometric analysis

Splenic mononuclear cells were passed through a 70-μm cell strainer, rinsed with phosphate-buffered saline, and then resuspended in 40 % Percoll solution (GE Healthcare, Chalfont St Giles, UK). The suspension was subjected to centrifugation at 2400 rpm for 30 min at a temperature of 4 °C. Subsequently, the cells were treated with fluorochrome-labeled monoclonal antibodies for a duration of 60 min at 4 °C. To exclude dead cells, anti-FVD-APC-Cy7 (eBioscience, San Diego, CA, USA) was included. The panel of antibodies used for staining comprised anti-CD3-PE-eF610, anti-PD-1-PE, anti-CD4-PerCP, anti-CD8-AF700, anti-CD62L-APC, and anti-CD44-FITC. For macrophage lineage staining, anti-CD45-A700, anti-CD11b-BV605, anti-F4/80-APC, and anti-MHC class II-PE-eF610 antibodies were used. To prevent non-specific antibody attachment, cells were initially treated with an anti-mouse CD16/32, which blocks mouse Fc receptors. (BD Biosciences, San Jose, CA, USA) Before applying specific antibodies for staining, bone marrow cells were pre-treated with a Cell Stimulation Cocktail (eBioscience, San Diego, CA, USA) to facilitate intracellular staining for 5 h. The cells were fixed and permeabilized using a Fixation/Permeabilization Buffer kit (eBioscience, San Diego, CA, USA), and then washed and resuspended in 1 % formaldehyde, and further stained for intracellular cytokines with anti-IFN-γ-APC, anti-TNF-α-PE antibody (eBioscience). Multicolor flow cytometry analysis was conducted using the LSRFortessa flow cytometer (BD Biosciences, NJ, USA), and the resulting data were processed and interpreted with the aid of FlowJo software (Tree Star, Ashland, OR, USA).

### 2.5 Cultivation and polarization of M1 macrophages

CD11b + bone marrow cells from femora of 8-week-old mice. The cells were seeded and cultured in DMEM medium supplemented with 10 % FBS, 1 % penicillin and 50 ng/ml M-CSF (PeproTech) for 6 days and treated with M1 cytokines (100 ng/mL LPS and 20 ng/mL IFN-γ) for an additional 24 h.

### 2.6 T cell viability assay

T cell viability was assessed using a Fixable Viability Dye (FVD) eFluor™ 780 (Invitrogen) according to the manufacturer's protocol. Following experimental treatments, CD4^+^ and CD8^+^ T cells were collected and washed twice with phosphate-buffered saline (PBS) to remove any residual media or serum proteins. Cells were resuspended in 1 mL of PBS and incubated with FVD at a final concentration of 1 μg/mL for 30 min at 4 °C in the dark. After incubation, the cells were washed twice with PBS to remove unbound dye. The stained cells were then resuspended in flow cytometry staining buffer (PBS containing 2 % fetal bovine serum and 2 mM EDTA) and kept on ice until analysis. Flow cytometric analysis was performed using the LSRFortessa flow cytometer (BD Biosciences). Dead cells were identified by their positive staining with FVD, as the dye reacts with free amines exposed in cells with compromised membrane integrity, resulting in a high fluorescence signal. Live cells, which exclude the dye, exhibited low fluorescence. Data were analyzed using FlowJo™ software (BD Biosciences), and the percentage of viable (FVD-negative) CD4^+^ and CD8^+^ T cells was calculated relative to the total cell population.

### 2.7 Single cell transcriptome analysis

A gene-cell matrix for single cell transcriptomics was downloaded from the GEO (GSE159977 and E-MTAB-10553) [15,16], and the output of the count matrix was read using the Read10X function in the Seurat package (Version 4.1.0) and the read.table function; the latter was further converted to dgCMatrix format. The outputs of the count matrix were read using the Read10X function from the Seurat package (Version 4.1.0) and the read.table function, and the latter was further converted to dgCMatrix format. After generating the feature-barcode matrix, we discarded cells with total unique molecular identifier count <500. To exclude low-quality cells from the data, we filtered out those in which mitochondrial genes accounted for >20 % of the total number. We excluded outlier cells from the downstream analysis using the isOutlier function in the scater R package. A global-scaling normalization method (“LogNormalize”) was employed to ensure that the total gene transcript expression in each of the cells was similar, and the scaling factor was set to 10,000. The top 2000 differentially expressed genes were subjected to downstream analysis using the FindVariableFeatures function. The ScaleData function, “vars.to.regress” option UMI, and percentage mitochondrial gene content were used to remove unwanted sources of variation.

Principal components analysis (PCA), incorporating highly variable features, was used to reduce the dimensionality of this dataset, and the first 30 PCs were identified for analysis. FindClusters using shared neighbor module optimization was then used, based on the first 17 PCs with a clustering resolution of 0.1, to create eight initial clusters. Most of the parameters we tried produced similar UMAP clustering, but the use of 17 PCs was associated with the best separation between different cell types. For each cell type, a marker gene was identified using the Seurat function FindAllMarkers and MAST. All analyses were performed in the Seurat R package (version 4.1.0) [16].

### 2.8 Measurement of mitochondrial oxygen consumption rate and extracellular acidification rate

The oxygen consumption rate (OCR) of the islets was measured using a Seahorse XF-24 according to the manufacturer's instructions (Seahorse Bioscience, North Billerica, MA, USA)18. The OCR was normalized to the mean baseline measurement in 2.8 mmol/l glucose and is expressed as a percentage change from baseline.

### 2.9 Statistical analysis

All Statistical analyses were conducted using GraphPad Prism software (version 9, Dotmatics, San Diego, USA). Data were expressed as the mean ± SD. Unpaired Student's t-tests and one-way ANOVA followed by Scheffe's post-hoc test were used to determine statistical significance, with a p-value of less than 0.05 considered significant. The ‘ggstatsplot’ creates graphics with details from statistical tests included in the plots themselves. Spearman's correlation was used to analyze the relationships between normally distributed variables. Statistical analyses were performed using R software version 4.1.0 (R project for Statistical Computing, Vienna, Austria), and P < 0.05 was considered to indicate statistical significance.

One-way analysis of variance (ANOVA) was used for multiple group comparisons with Tukey's posthoc test when data were normally distributed. The analyses were performed using GraphPad-Prism 7.0 (GraphPad Software, Inc., San Diego, CA). Significant differences were determined statistically at *p <* 0.05.

## 3 Results

### 3.1 RGE increases mitochondrial function of CD4^+^ T cells from young and old mice

**Fig. 1:**
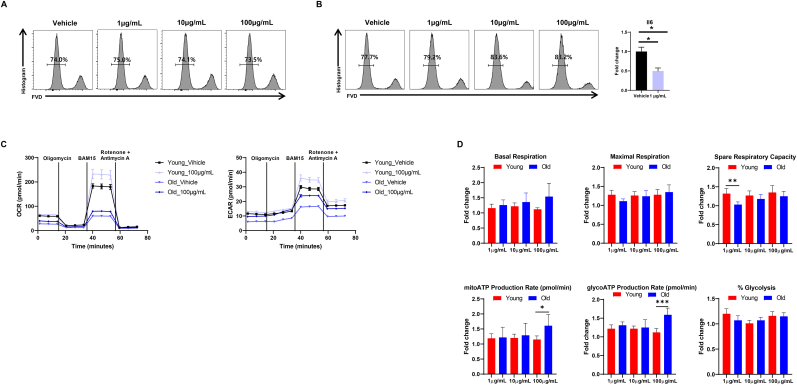
Red ginseng extract enhances mitochondrial function of CD4^+^ T cells from young and aged mice. (A, B) Cell viability assay for young (12-week-old) and aged (20-month-old) CD4^+^ T cells treated with or without red ginseng extract using fixable viability dyes. (C, D) Oxygen consumption rate (OCR) and extracellular acidification rate (ECAR) (left panel) and individual parameters (right panel) in CD4^+^ T cells isolated from young and old mice. The cells were treated with oligomycin (2 μg/mL), CCCP (10 μM), or rotenone (1 μM). ∗P < 0.05, ∗∗P < 0.01, and ∗∗∗P < 0.001.

To define the effect of RGE on mitochondrial function in T cells, we isolated and cultured primary CD4^+^ T cells from 2-month-old and 20-month-old mice. The treatment with 1 μg/mL of RGE resulted in no significant change in cell viability of CD4^+^ T cells isolated from 2-month-old mice (Fig. 1A), but 10 and 100 μg/mL of RGE treatment enhanced live cell frequencies of the CD4^+^ T cells (Fig. 1B). The cellular viability of CD4^+^ T cells isolated from 20-month-old mice were not affected by RGE treatment, indicating that RGE does not negatively impact the survival of CD4^+^ T cells regardless of age in mice. Furthermore, RGE treatment led to a marked increase in mitochondrial OCR in the CD4^+^ T cells from both 2-month-old and 20-month-old mice (Fig. 1C), suggesting an enhancement in mitochondrial respiration. We calculated the fold change for each concentration based on the Vehicle of young and old groups, respectively, and examined the changes in OCR at each concentration between young and old groups. As a result, we observed that basal respiration was increased in old compared to young, and at a concentration of 100 μg/mL, the changes in both mitoATP production rate and glycoATP production rate were higher in old than in young. (Fig. 1 D). In parallel, there was a notable increase in the glycolytic capacity of CD4^+^ T cells (Fig. 1C and D), demonstrating that RGE promotes both mitochondrial and glycolytic metabolic pathways in these cells. Moreover, the changes induced by RGE were more pronounced in the old group.

### 3.2 RGE increases mitochondrial function of CD8^+^ T cells from young and old mice

**Fig. 2:**
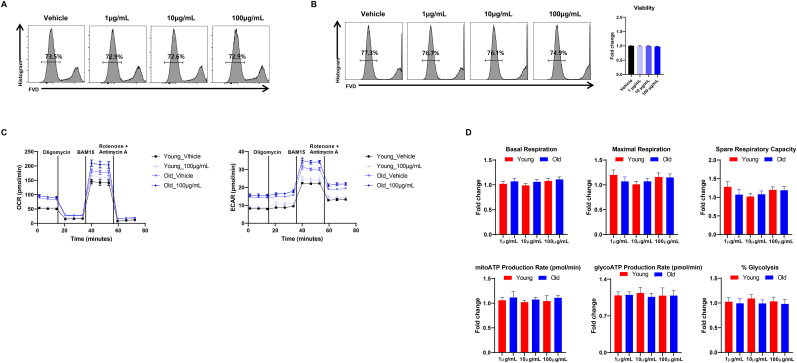
Red ginseng extract modestly improves mitochondrial function of CD8^+^ T cells from young and aged mice. (A, B) Cell viability assay for young (12-week-old) and aged (20-month-old) CD8^+^ T cells treated with or without red ginseng extract using fixable viability dyes. (C, D) Oxygen consumption rate (OCR) and extracellular acidification rate (ECAR) (left panel) and individual parameters (right panel) in CD8^+^ T cells isolated from young and old mice. The cells were treated with oligomycin (2 μg/mL), CCCP (10 μM), or rotenone (1 μM). Data are expressed as means ± standard deviation. ∗P < 0.05, ∗∗P < 0.01, and ∗∗∗P < 0.001.

Aging increases systemic circulating CD8^+^ T cells in mice and humans. The reduction in absolute numbers of naïve CD8^+^ T cells and the increase in memory CD8^+^ T cells are key hallmarks of immune aging. Thus, we evaluated whether RGE treatment has an impact on mitochondrial function of young and old CD8^+^ T cells. RGE treatment similarly influenced CD8^+^ T cells, showing either no significant effect on viability or an increase in cell survival, akin to the effects observed in CD4^+^ T cells from 2-month-old and 20-month-old mice (Fig. 2A and B). Notably, RGE enhanced both OCR and glycolytic capacity in CD8^+^ T cells from 2-month-old and 20-month-old mice (Fig. 2C). However, we calculated the fold change values for CD8^+^ T cells and compared young and old groups. As a result, we found that the changes in OCR parameters induced by RGE treatment were not significantly different between young and old in CD8^+^ T cells. (Fig. 2C and D). Moreover, RGE treatment enhanced both OCR and glycolytic capacity in LPS-induced M1 polarized macrophages (Supplemental Fig. 1A and B). During the M1 polarization of bone marrow derived macrophages, the mRNA expression of M1 macrophages makers, including *Il6*, *Tnf*, *Il1β*, *Ccl2* was significantly suppressed by RGE (Supplemental Fig. 1C), suggesting that RGE's impact on metabolic function is more substantial in T cells and macrophages.

### 3.3 RGE attenuates T cell senescence and exhaustion in mice

**Fig. 3:**
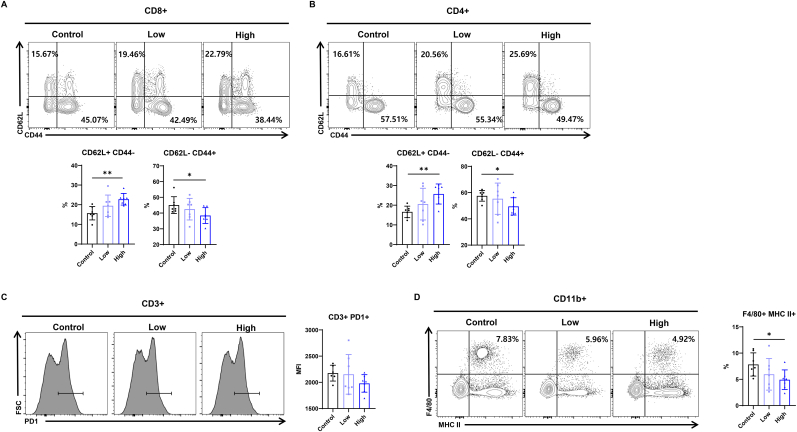
Red ginseng extract attenuates proinflammatory cytokine-producing T cells in aged mice. (A, B) Population size and frequency of CD44+CD62L− and CD44^−^CD62L+ in CD4^+^, and CD8^+^ T cells in liver tissues of 20‐month‐old mice treated with or without red ginseng extract. (C) Representative histogram flow cytometry plots and MFI statistical analysis of PD-1+ mature T cells in liver tissues of 20‐month‐old mice treated with or without red ginseng extract. (D) Percentage of MHCII+ and F4/80+ cells in CD11b + myeloid cells within the livers of 20‐month‐old mice treated with or without red ginseng extract. Data are expressed as means ± standard deviation. ∗P < 0.05 and ∗∗P < 0.01.

**Fig. 4:**
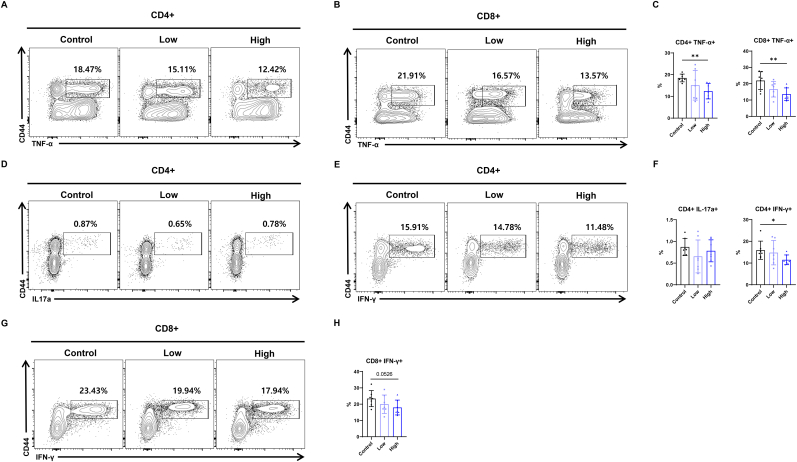
Red ginseng extract reduces proinflammatory cytokine-producing T cells in aged mice. (A–C) TNF‐α-producing CD4^+^ and CD8^+^ T cells in liver tissues of 20‐month‐old mice treated with or without red ginseng extract. (D–F) Population size of IL-17A- and IFN-γ-producing CD4^+^ T cells in liver tissues of 20‐month‐old mice treated with or without red ginseng extract. (G, H) IFN-γ-producing CD8^+^ T cells in liver tissues of 20‐month‐old mice treated with or without red ginseng extract. Data are expressed as means ± standard deviation. ∗P < 0.05 and ∗∗P < 0.01.

Based on *in vitro* findings suggesting RGE's potential effects on mitochondria of the T cells, we conducted in vivo experiments to further validate RGE's role in modulating T cell function in 20-month-old mice (Supplemental Fig. 2A). Splenic mononuclear cells were first gated for single cells (forward scatter-area vs. forward scatter-height) and lymphocytes (forward scatter-area vs. side scatter-area). The lymphocyte population was then further analyzed for uptake of a fixable viability dye to determine the proportion of live cells, and stained for CD3. The surface expression of CD4 and CD8 was then determined in this gated population (Supplemental Fig. 2B). *In vivo* experiments demonstrated that 20-month-old mice administered with RGE exhibited a significant increase in the proportion of naive CD4^+^ and CD8^+^ T cells, along with a corresponding decrease in memory T cells, compared to the control group (Fig. 3A and B). Analysis of PD-1 expression based on MFI values revealed that PD-1 expression decreased as the concentration of RGE increased (Fig. 3C). Additionally, RGE administration led to a reduction in MHC class II expression on macrophages (Fig. 3D). Furthermore, RGE-treated mice showed reduced expression of IFN-γ and TNF-α in both CD4^+^ and CD8^+^ T cells, as well as a decrease in IL-17-producing CD4^+^ T cells (Fig. 4A–H). These findings suggest that RGE may modulate the immune system by promoting the maintenance of a naive T cell pool, reducing pro-inflammatory cytokine production, and downregulating MHCII expression on macrophages. This shift in immune cell populations and cytokine expression indicates a potential role of RGE in suppressing systemic inflammation, particularly by mitigating age-associated inflammatory responses.

### 3.4 Single cell transcriptome analysis indicates RGE-mediated improvements of T cell senescence and mitochondrial function

**Fig. 5:**
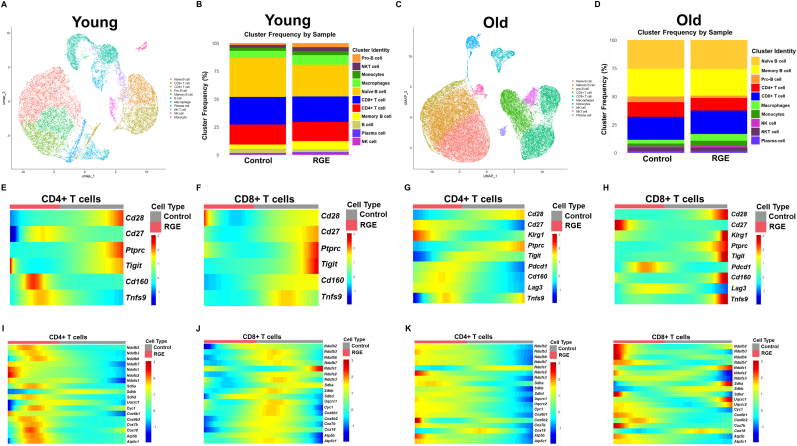
Single cell transcriptomics data analysis of young and old mice treated with low concentration RGE. Spleen samples were analyzed to characterize the immune population changes according to RGE. Approximately 21,562 and 28,919 single cells were extracted from the young and old mice groups, respectively, and utilized for single cell RNA sequencing analysis. (A) In young mice group, 2D UMAP representation showing the cells from controls and RGE treated mice and their assignment 11 clusters by Leiden clustering. (B) Bar plot showing the relative cluster ratio of immune cells from control and RGE in young mice. (C) 2D UMAP representation showing control and RGE cells in old mice jointly and their assignment 10 clusters by Leiden clustering. (D) Bar plot showing the relative cluster ratio of immune cells from control and RGE in old mice. (E, F) Pseudotime heatmap showing senescence gene expression of systemic CD4^+^ and CD8^+^ T cells from control and RGE in young mice. (G, H) Senescence gene expression of systemic CD4^+^ and CD8^+^ T cells from control and RGE in old mice, analyzed with pesudotime heatmap. Pseudotime heatmap showing mitochondrial OXPHOS complex genes of systemic CD4^+^ and CD8^+^ T cells from control and RGE in young mice (I, J), and old mice (K, L).

**Fig. 6:**
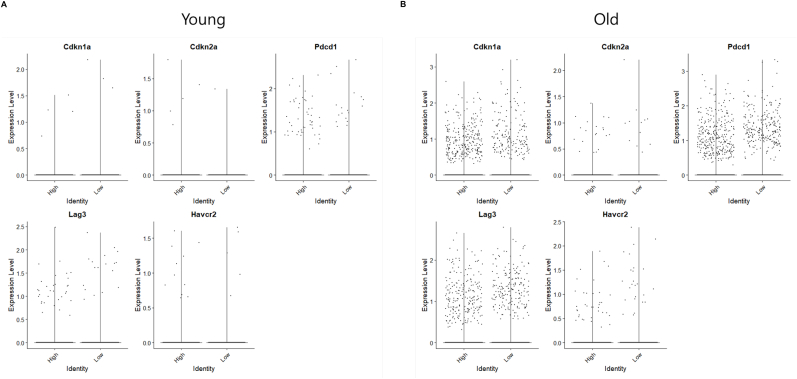
Expression of immunosenescence-associated genes in T cells with high vs. low OXPHOS activity after RGE treatment. T cells from RGE-treated young (A) and old (B) mice were stratified into “High” and “Low” groups based on OXPHOS gene set expression. Expression levels of sene scence-related markers (*Cdkn1a*, *Cdkn2a*) and exhaustion markers (*Pdcd1*, *Lag3*, *Havcr2*) were compared between the groups.

To elucidate the in vivo effects of RGE on T cell senescence and mitochondrial function, we performed single cell transcriptome analysis on splenocytes isolated from both young and old mice following RGE treatment. First, we integrated the datasets from old and young mice and performed clustering based on the expression of specific cell markers to assess how the clusters change during aging. We further observed that, following RGE treatment, only T cell and macrophage markers exhibited specific changes, while other immune cell markers remained largely unaffected. (Supplemental Fig. 3A–C). We observed a marked increase in the expression of senescence markers and pro-inflammatory markers in the old group compared to the young group (Supplemental Fig. 3D). Subsequently, we conducted separate analyses for the young and old groups. In young mice, UMAP analysis identified distinct immune cell populations, including CD4^+^, CD8^+^, NKT cells, NK cells (lymphocytes), monocytes, macrophages, and plasma cells along with various B cell types (Fig. 5A). In case of the treatment with RGE, there was an increase in the NKT cell population and a decrease in CD4^+^ and CD8^+^ T cells (Fig. 5B). A similar immune profile was observed in old mice group (Fig. 5C), but no significant changes in NKT cells, while CD4^+^ and CD8^+^ T cells decreased after RGE treatment (Fig. 5D). Upon comparison of the young and old groups, with and without RGE treatment, no significant changes were observed in ROS levels or in the expression of genes associated with mitochondrial fitness (Supplemental Fig. 4A–E). Nevertheless, the expression of OXPHOS complex genes was found to be markedly reduced in the old group (Supplemental Fig. 4F–H). In light of these observations, pseudotime analysis was subsequently performed to investigate gene expression dynamics along the pseudotime trajectory between the control and RGE-treated groups. As a result of the pseudotime analysis, we observed that in the RGE treatment group, the expression of senescence markers such as *Cd28* and *Cd27* decreased in both CD4^+^ and CD8^+^ T cells from young, as the pseudotime progressed from the control to the condition of RGE treatment (Fig. 5E–H). Single cell transcriptome analysis revealed that the expression of senescence-related genes modestly decreased in response to RGE treatment in both young and old mice. This reduction was observed across various cell types, except for CD8^+^ T cells in young mice (Supplemental Figs. 5 and 6). Furthermore, we investigated the changes in oxidative phosphorylation (OXPHOS) complex, which plays an important role in T cell mitochondrial function. Upon examining the expression of genes in each OXPHOS complex, we observed a significant increase in the expression of *Ndufb2*, *Sdha*, *Uqcrc1*, *Cox6b2*, and *Atp5b*, which are representative of the OXPHOS complexes, in the RGE treatment group, except for the CD8^+^ T cells of young mice. (Fig. 5I–L). Bar plot analysis also showed that the expression of OXPHOS complex genes increased in CD8^+^ T cells from both young and old mice following RGE treatment (Supplemental Figs. 5 and 6). The analysis revealed distinct transcriptional signatures that highlight the impact of RGE on key pathways involved in T cell senescence and mitochondrial dynamics, offering new perspectives on its potential therapeutic benefits in modulating immune aging. Next, to investigate whether the RGE-induced increase in OXPHOS contributes to the suppression of T cell immunosenescence, we divided both young and old mice into high and low groups based on the expression levels of the OXPHOS gene set after RGE treatment and compared the expression of immunosenescence marker genes between the groups. To determine whether increased OXPHOS activity contributes to the suppression of T cell immunosenescence, we stratified RGE-treated T cells into high and low OXPHOS gene set expression groups and compared the expression of senescence-associated markers. In young mice, there were no substantial differences in the expression levels of *Cdkn1a*, *Cdkn2a*, *Pdcd1*, *Lag3*, or *Havcr2* between the high and low OXPHOS groups. However, in old mice, cells with higher OXPHOS activity exhibited a clear reduction in the expression of these immunosenescence markers, including *Cdkn1a*, *Cdkn2a*, *Pdcd1*, *Lag3*, and *Havcr2*. These findings suggest that the upregulation of OXPHOS in aged T cells may be functionally linked to the attenuation of senescence-associated gene expression following RGE treatment (Fig. 6).

### 3.5 RGE treatment attenuates macrophage-mediated T cell activation and reduces immune senescence

**Fig. 7:**
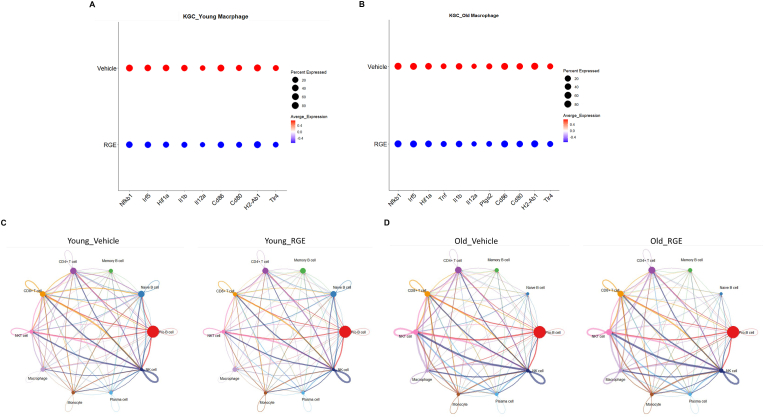
Cell-cell communication networks among immune cell populations in vehicle- and RGE-treated mice. (A, B) Dot plots show the expression patterns of representative pro-inflammatory and activation-related genes in macrophages isolated from old (left) and young (right) mice. (C) Circle plots show the inferred intercellular signaling interactions based on ligand-receptor expression profiles from single-cell transcriptome data. (Left) vehicle-treated and (Right) RGE-treated groups are shown for young mice. (D) Circle plots show the inferred intercellular signaling interactions based on ligand-receptor expression profiles from single-cell transcriptome data. (Left) vehicle-treated and (Right) RGE-treated groups are shown for old mice.

Next, to investigate the mechanism by which RGE suppresses T cell senescence, we conducted a single-cell analysis. We observed that macrophage activation was reduced in both young and old mice following RGE treatment (Fig. 7A and B). To further examine how these changes in macrophage activation affect T cell activation, we performed a cell-cell communication analysis. Cell-cell communication analysis demonstrated that macrophages exhibited robust interactions with both CD4^+^ and CD8^+^ T cells in the vehicle-treated groups of both young and old mice. These strong signaling pathways, visualized as thick directional edges, indicate active immune communication and potential pro-inflammatory activation of T cells. However, following RGE treatment, the macrophage-to-T cell interactions were markedly reduced in both age groups. This consistent decrease across young and old mice suggests that RGE treatment attenuates macrophage-driven activation of T cells, potentially contributing to reduced immune senescence and inflammation (Fig. 7C and D).

## 4 Discussion

In this study, RGE significantly enhances mitochondrial OCR of CD4^+^ and CD8^+^ T cells from both young and aged mice. LPS-induced M1 polarization in macrophages was remarkably attenuated by RGE treatments accompanied by increased mitochondrial function. *In vivo* experiments revealed that treatment with RGE alleviates T cell senescence and exhaustion in mice. Single cell transcriptome analysis using splenocytes suggests that RGE-mediated improvements of T cell senescence and exhaustion are linked to an increase in the expression of geneset of mitochondrial function in the T cells.

T cell aging is a critical area of research due to its profound implications for immune system function and overall health during aging. As individuals age, T cells undergo functional declines, including reduced proliferative capacity, altered cytokine production, and impaired response to pathogens and vaccines [1,[4], [5], [6]]. This immunosenescence contributes to increased susceptibility to infections, reduced vaccine efficacy, and a higher incidence of age-related diseases, such as metabolic diseases and cancers [[13], [14], [15], [16], [17]]. Moreover, the aging of T cells is associated with chronic inflammation, often termed "inflammaging," which is a key driver of various age-related pathologies [7,[17], [18], [19]]. Understanding the metabolic reprogramming underlying inflammating and aging in T cells is essential for augmenting host defense strategies to enhance immune function in the elderly, potentially improving healthspan and quality of life [14]. As T cells age, mitochondrial dysfunction manifests through increased reactive oxygen species (ROS) production, impaired oxidative phosphorylation, and altered mitochondrial dynamics, all of which contribute to cellular senescence [20]. These mitochondrial defects can lead to reduced T cell proliferative capacity, diminished effector functions, and increased susceptibility to apoptosis [15,21]. Activated T cells from older adults contribute to inflammaging by releasing mitochondrial DNA (mtDNA) into their environment [22]. T cells with mitochondrial dysfunction caused by a deficiency in mitochondrial transcription factor A (TFAM) accelerate the process of senescence [21]. The mitochondrial Ca2+ uptake capacity of mouse macrophages significantly decreases with age, and restoring this capacity in tissue-resident macrophages may help reduce inflammaging [23]. Mice with myeloid-specific mitochondrial dysfunction, leading to reduced mitochondrial oxidative phosphorylation (OxPhos), exhibit M1-like macrophage polarization, systemic insulin resistance, and inflammation in adipose tissue [24]. Therefore, targeting mitochondrial pathways offers a promising strategy to mitigate T cell aging, potentially improving immune resilience and reducing age-related disease burden.

This study indicates that the potential immunomodulatory effects of RGE extend beyond its anti-inflammatory properties, particularly in the context of immune aging and inflammaging. RGE enhances mitochondrial function in both T cells and macrophages, which are essential for maintaining immune balance. During aging process, immune cells tend to exhibit nonspecific activation due to mitochondrial dysfunction, contributing to augmentation of proinflammatory senescence-associated secretory phenotype, which leads to immune aging and chronic low-grade inflammation, known as inflammaging [25]. By improving mitochondrial function, RGE could help counteract these age-related changes, reducing the nonspecific activation of immune cells. These effects would position RGE as a potential therapeutic agent in managing age-related immune dysregulation, offering benefits in both reducing inflammaging and maintaining immune homeostasis.

In support of this, our single-cell transcriptome analysis revealed that RGE treatment modulates immune cell metabolism and intercellular signaling in a manner that alleviates immunosenescence, particularly in aged mice. In T cells from old mice, elevated expression of OXPHOX complex gene sets were associated with significantly reduced levels of senescence- and exhaustion-associated markers, including *Cdkn1a*, *Cdkn2a*, *Pdcd1*, *Lag3*, and *Havcr2*. In contrast, this association was not observed in young mice, suggesting that the impact of mitochondrial enhancement is age-dependent. Furthermore, cell-cell communication analysis showed that macrophage-derived signaling toward CD4^+^ and CD8^+^ T cells was markedly reduced following RGE treatment. This was accompanied by decreased expression of pro-inflammatory genes such as *Tnf*, *Il1b*, *Cd80*, and *Cd86* in macrophages. Together, these results highlight a dual mechanism by which RGE mitigates immunosenescence—through direct metabolic reprogramming of T cells and attenuation of macrophage-driven inflammatory signaling.

The strength of this study was the showing RGE's potentials on the anti-aging of immune cells *in vitro* and in vivo. Moreover, single cell transcriptome analysis using splenocytes suggested the genesets of mitochondrial function as a potential candidate mechanism of the reversing aging of the immune cells. However, this study has several limitations. First, there was no experiment conducted to directly evaluate whether the administration of RGE restores immune function decline caused by aging. Additionally, we did not propose the candidate molecular mechanism by which mitochondrial function improvement occurred due to RGE treatment in T cells and macrophages even though RGE remarkably attenuates proinflammatory cytokine production in the immune cells of the aged mice. Although previous investigations indicate that ginsenosides possess antioxidant, anti-inflammatory, and immunomodulatory properties [26], further research is needed to analyze the effects of individual components of RGE on various immune cells to determine which component contributed to the improvement of mitochondrial function. Furthermore, it is necessary to attempt to demonstrate the anti-aging effects of RGE by utilizing various indicators of immune cell aging.

The active ingredients that make up red ginseng are very diverse. These ingredients have different special ingredients that are activated to normalize external stimuli and biological changes. In this paper, the ingredients related to the T cell immune aging alleviation effect identified by the research team are predicted to be ginsenoside Rg1 and Rb1 in the saponin fraction derived from Korean red ginseng, and polysaccharides are expected to be involved in the non-saponin fraction [27]. showed that ginsenoside Rg1, when administered intraperitoneally and intravenously to mice, enhances immunity by increasing the number of antigen-reactive T cells. They also revealed that Rg1 increases the number of T helper cells relative to the total number of T cells and promotes the activation of natural killer cells in splenocytes. Also, in the reviewed paper, You L et al. [28] are claims that ginsenoside Rg1 has immunomodulatory roles in immune system in-vitro and in-vivo.

And another previous study confirmed that oral administration of ginsenoside Rb1 to mice alleviated DON-induced immune damage, and confirmed that this was due to the enhancement of antioxidant capacity and inhibition of excessive apoptosis through regulation of the mitochondrial apoptosis pathway. In addition, we confirmed an increase in the ratio of CD4^+^ and CD8^+^ T cells, providing a therapeutic effect of Rb1 on the immune function of damaged mice [29].

In the non-saponin fraction [30], conducted research about Immune Activity of Polysaccharide Fractions Isolated from Korean Red Ginseng. They showed that the intraperitoneal macrophage phagocytosis activity and the number of T cells, B cells, and macrophages in the spleen increased significantly by oral administration of Polysaccharide Fractions from Korean Red Ginseng.

In previous research, the tolerability and pharmacokinetic properties of ginsenosides, including Rb1, were elucidated after single or multiple administrations of red ginseng extract to humans [31]. Pharmacokinetic parameters of ginsenoside Rb1 after single administration of red ginseng extract are Tmax(h)_3.53 ± 1.25, Cmax(ng/mL)_2.19 ± 0.73, AUC(ng∗h/mL)_ 82.68 ± 26.26 and repeated administration of red ginseng extract are Tmax(h)_2.12 ± 1.76, Cmax(ng/mL)_12.20 ± 7.34, AUC(ng∗h/mL)_ 375.93 ± 128.17. And Rg1 belongs to the PPT group, pharmacokinetic parameters of PPT after receiving red ginseng in healthy volunteers [32] are Tmax(h)_19.04, Cmax(ng/mL)_3.50 ± 2.00, AUC24(ng∗h/mL)_ 34.11 ± 24.68, AUClast(ng∗h/mL)_45.74 ± 32.10.

Additionally, the relationship between absorption rates between polysaccharides [33] and ginsenosides was also studied. Research reported that ginseng polysaccharides can promote the absorption of ginsenosides to effect the microbial environment in the gut of rats [34] and polysaccharides increase the systemic exposure of Rb1 [35]. But, absorption rate of polysaccharides are unclear. The relationship between the absorption rates of red ginseng ginsenosides and polysaccharides can be used to predict the immune aging improvement effects of Rg1, Rb, and polysaccharides.

In conclusion, our findings demonstrate the efficacy of RGE on mitochondrial function *in vitro* and in vivo, highlighting its potential as a novel treatment for aging-associated inflammaging. The ability of RGE to modulate mitochondrial function in T cells and macrophages, along with its impact on immune aging, suggests that targeting mitochondria in immune cells may be a promising avenue for developing effective therapies for aging-mediated inflammatory disorders.

## Funding

This work was supported by the Basic Science Research Program, through the 10.13039/501100003725National Research Foundation of Korea (10.13039/501100003725NRF), funded by the Ministry of Science, ICT, and Future Planning, Korea (NRF-RS-2024-00349240, NRF-2021R1A5A8029876, and NRF-2023R1A2C3006220). H.S.Y. was supported by a grant from the Korea Health Technology R&D Project, through the 10.13039/501100003710Korea Health Industry Development Institute (10.13039/501100003710KHIDI), funded by the Ministry of Health & Welfare, Republic of Korea (grant number: RS-2022-KH130308 and RS-2024-00507183) and supported by the PRIDE research institute funding program at 10.13039/501100002462Chungnam National University.

## Conflict of interest

S.H.L. and H.K.K. are employees of Korea Ginseng Corp., a leading company that specializes in producing popular Korean Red Ginseng products and conducting extensive research on the safety of raw materials to promote human health. The other authors declare no competing interests.
