# Supplementary material for: Red ginseng extract enhances mitochondrial function and alleviates immunosenescence in T cells
Source: J Ginseng Res. 2025 May 20;49(5):564–73. doi: 10.1016/j.jgr.2025.05.004 (PMC12365556; doi:10.1016/j.jgr.2025.05.004)
Supplement: Multimedia component 1 [file mmc1.docx]

***Journal of Ginseng Research***

***Original article***

- **Red ginseng extract enhances mitochondrial function and alleviates immunosenescence in T cells**
- Ho Yeop Lee^1,2,#^, Jingwen Tian^1,2,#^, Ha Thi Nga^1,2^, Thi Linh Nguyen^1,2^, Ji Sun Moon^1^, Hyo Ju Jang^1,2^, Alfin Mohammad Abdillah^1^, Si-Eun Lee^1^, Sang Hyeon Ju^4^, Seung Ho Lee^5^, Hun Kun Ko^5^, Minho Shong^4,^*, Hyon-Seung Yi^1,2,3,^*
- ^1^Laboratory of Endocrinology and Immune System, Chungnam National University School of Medicine, Daejeon, South Korea
- ^2^Department of Medical Science, Chungnam National University School of Medicine, Daejeon, South Korea
- ^3^Department of Internal Medicine, Chungnam National University School of Medicine, Daejeon, South Korea

^4^Graduate School of Medical Science and Engineering, Korea Advanced Institute of Science and Technology, Daejeon, South Korea

^5^R&D Headquarters, Korea Ginseng Corp., Gyeonggi-do, South Korea

Word Count: 3803 (excluding abstract, references, tables and figure legends)

Running title: Role of RGE in immunosenescence

***Correspondence to:**

HSY (jmpbooks@cnu.ac.kr); Tel: +82-42-280-6801; Fax: +82-42-280-6990

MS (minhos@kaist.ac.kr)


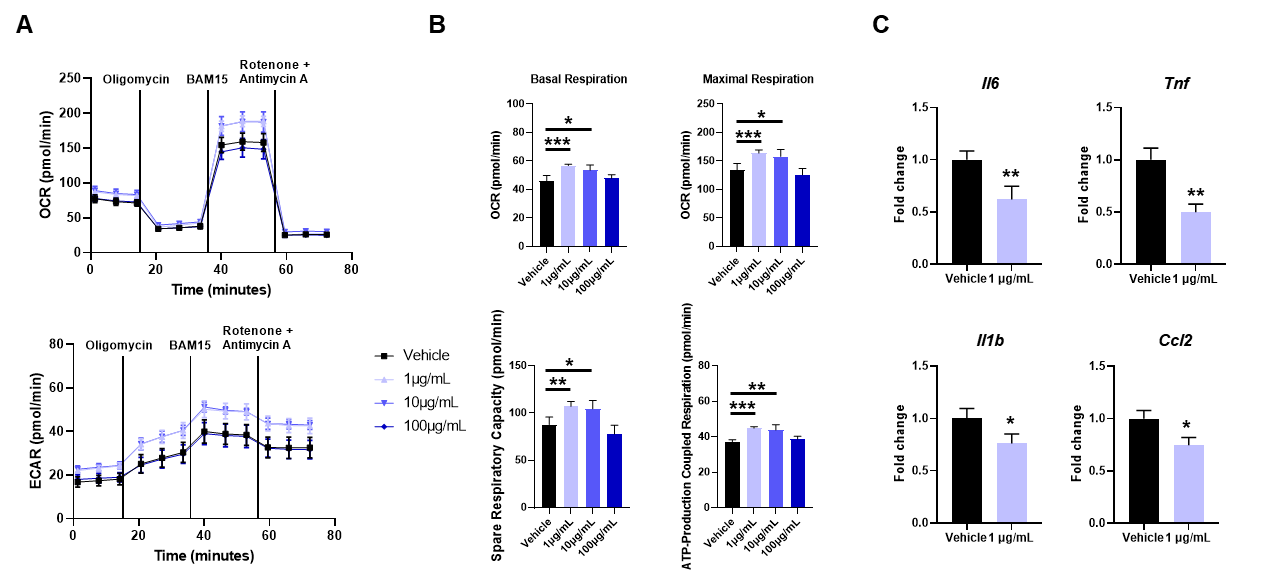


Supplemental Fig. 1. RGE effects on metabolic function in macrophages. (A, B) Oxygen consumption rate (OCR) and extracellular acidification rate (ECAR) in RGE treated macrophages isolated from young mice. The cells were treated with oligomycin (2 μg/mL), CCCP (10 μM), or rotenone (1 μM). (C) Relative expression of mRNA encoding genes related to M1 macrophage markers from M1 polarized macrophages in young mice. Data are expressed as means ± standard deviation. *P < 0.05, **P < 0.01, and ***P < 0.001.


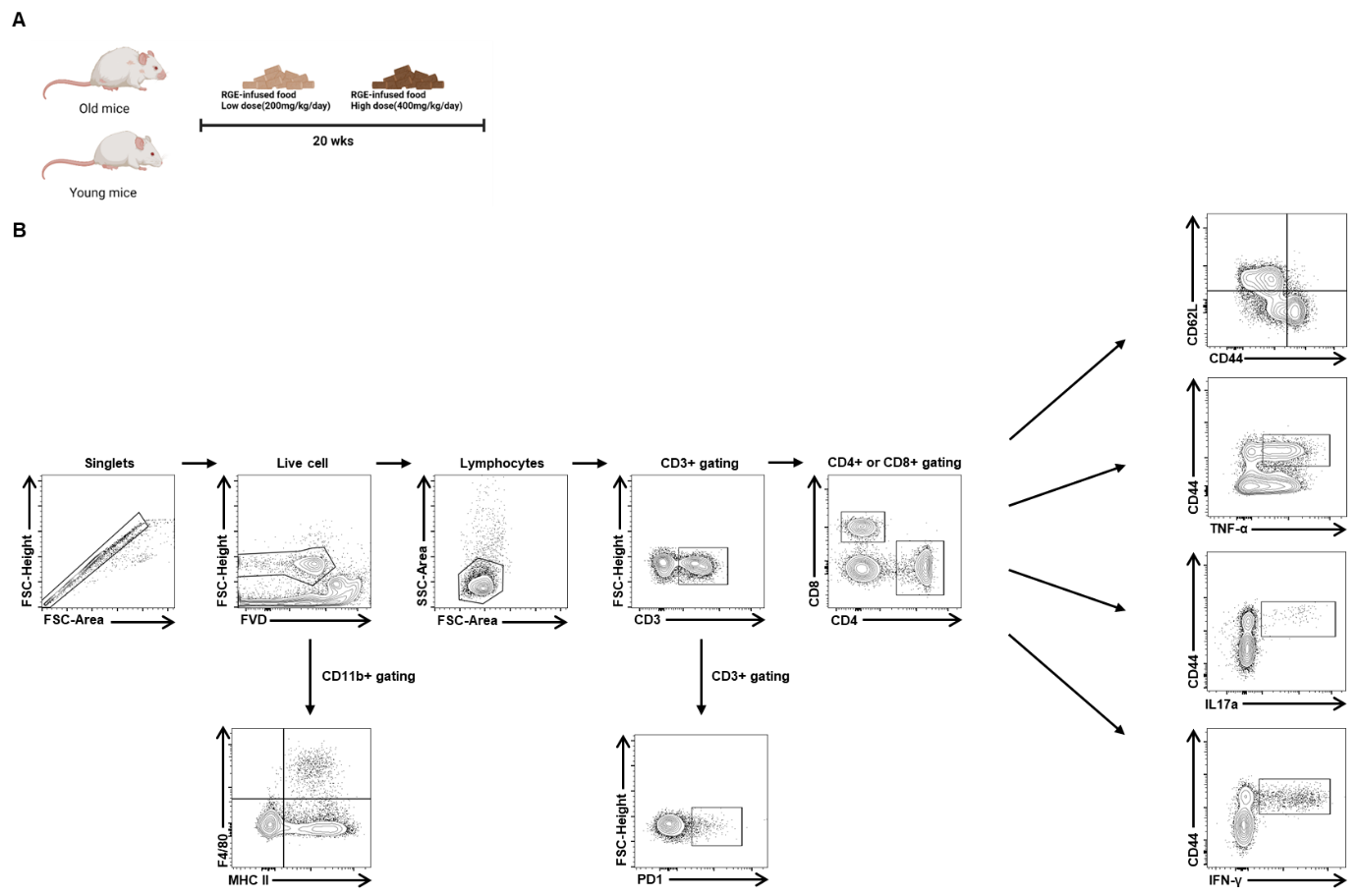


Supplemental Fig. 2. RGE feeding plan and flow cytometry strategy. (A) Schematic image for RGE feeding in young and old mice during 20 wks. (B) Gating strategy used for analysis of CD4+, CD8+ T cells and macrophage in liver of RGE treated young and old mice.


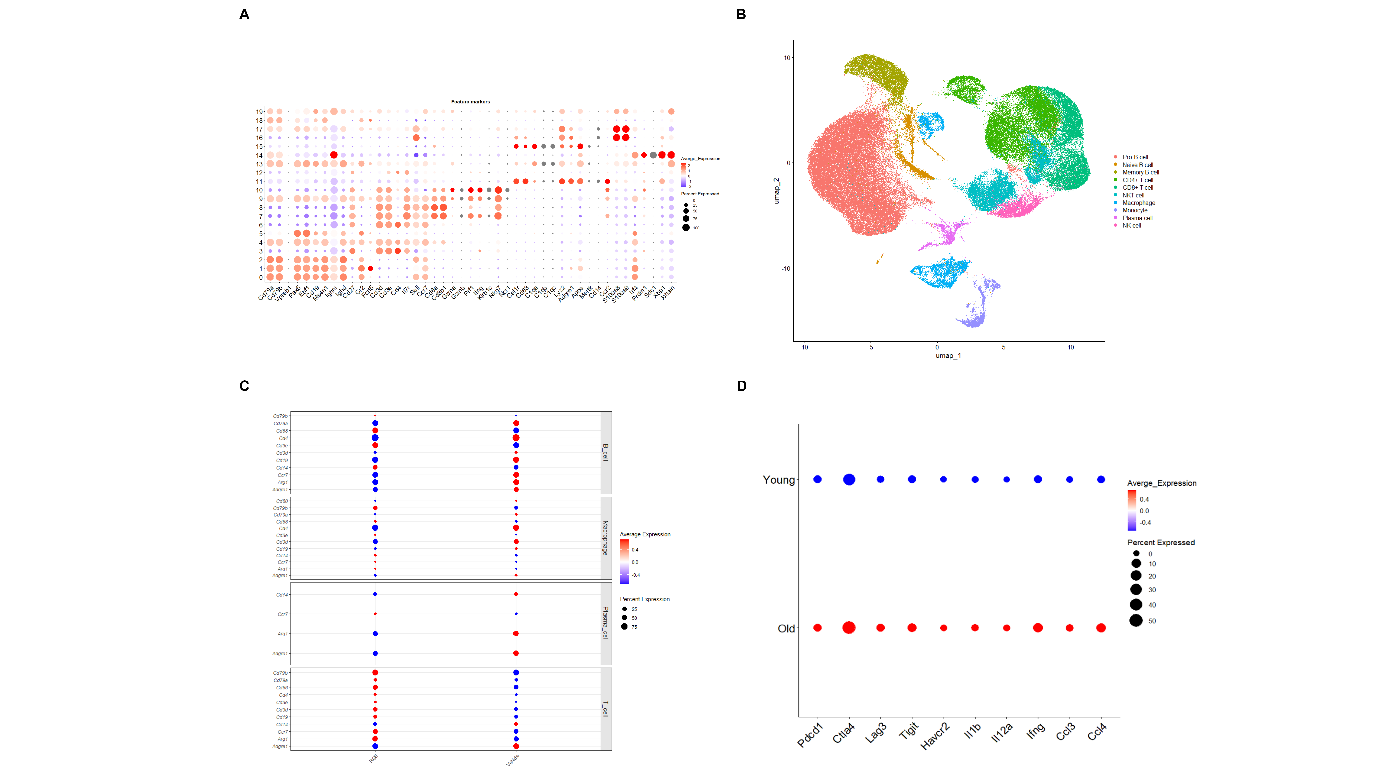


Supplemental Fig 3. Single-cell RNA-seq analysis of splenic immune cells from young and old mice with or without RGE treatment. (A) Dot plot showing the expression of representative market gene in young and old mice. (B) UMAP visualization of integrated single cell dataset from young and old mice. (C) Comparative dot plot analysis showing the expression of key B cell, macrophage, Plasma cell and T cell in control and RGE-treated groups from young and old mice. (D) Dot plot analysis of senescence and inflammation related gene expression in T cells from young and old mice.


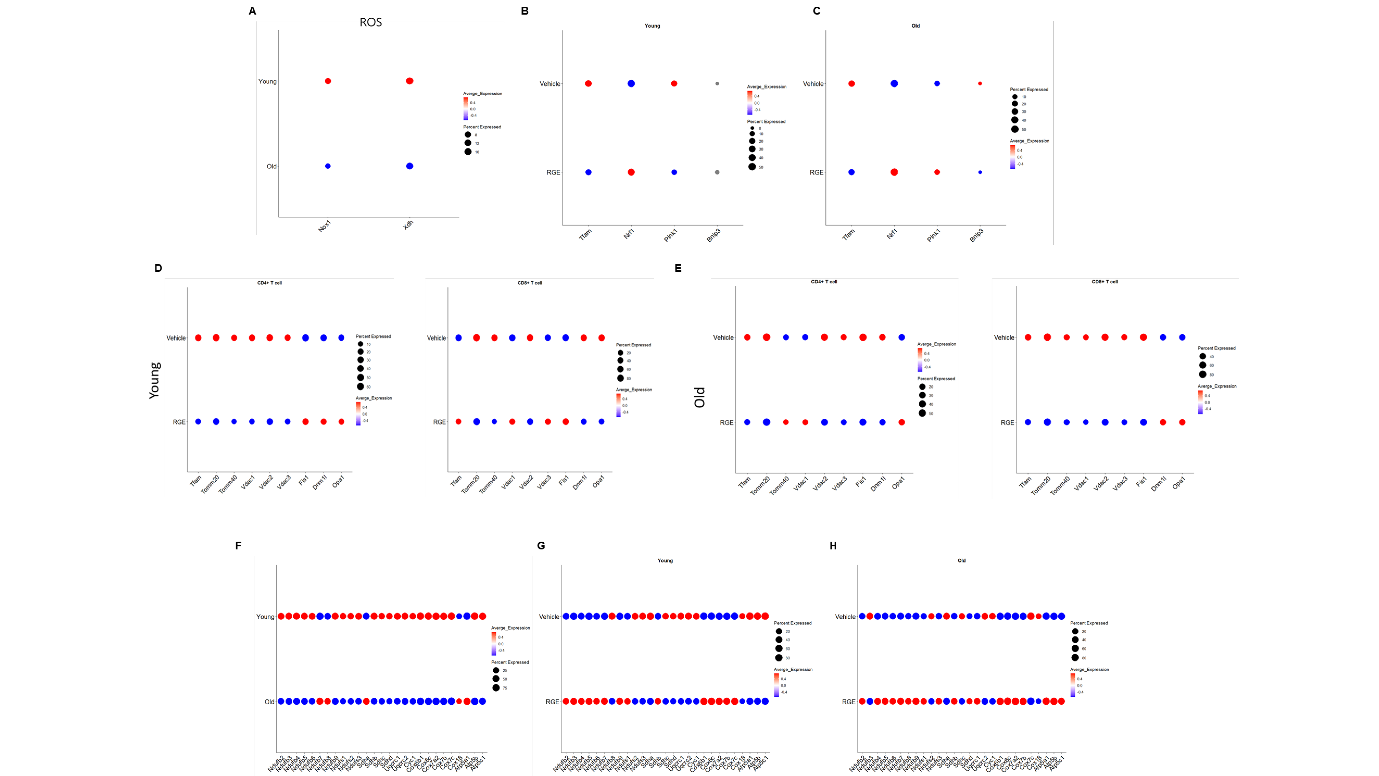


Supplemental Fig 4. Effects of RGE treatment on ROS-related genes, mitochondrial function, and OXPHOS gene expression in T cells from young and old mice. (A) Dot plot analysis of ROS-associated gene expression in splenic immune cells from young and old mice single cell datasets. (B, C) Expression of genes related to mitochondrial function (Tfam, Nrf1, Ppargc1a, Sirt3) in young (B) and old (C) mice. (D, E) Dot plots showing the expression of OXPHOS complex genes in CD4⁺ and CD8⁺ T cells under vehicle and RGE-treated conditions. (F-H) Comparative analysis of OXPHOS gene expression profiles between young and old groups following vehicle or RGE treatment


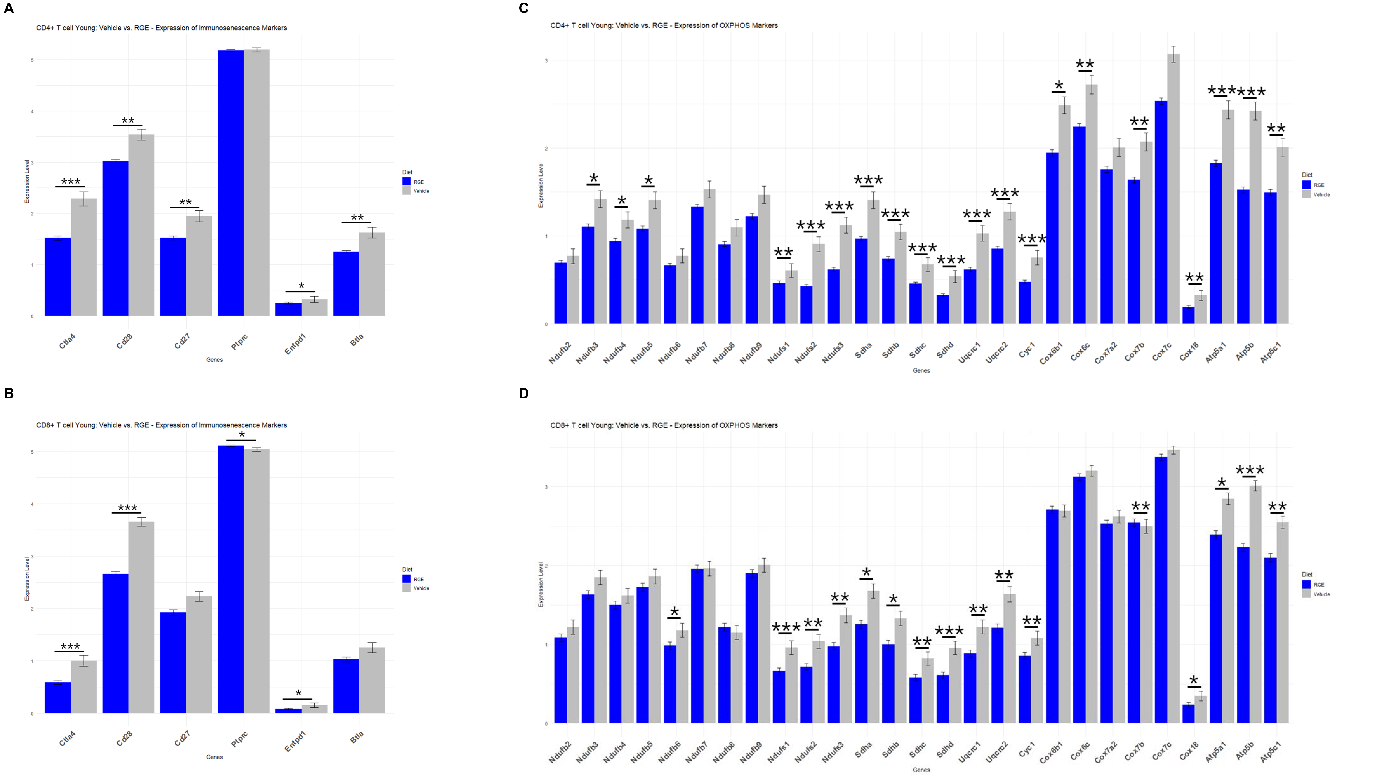


Supplemental Fig. 5. Metabolic function in CD4+ and CD8+ T cells from RGE treated young mice. (A, B) Bar plot of T cell senescence marker in CD4+ T cell and CD8+ T cell using RGE treated young mice single cell transcriptomics. (C, D) Bar plot of T cell OXPHOS complex marker in CD4+ T cell and CD8+ T cell using RGE treated young mice single cell transcriptomics. (Data are expressed as means ± standard deviation. *P < 0.05, **P < 0.01, and ***P < 0.001.


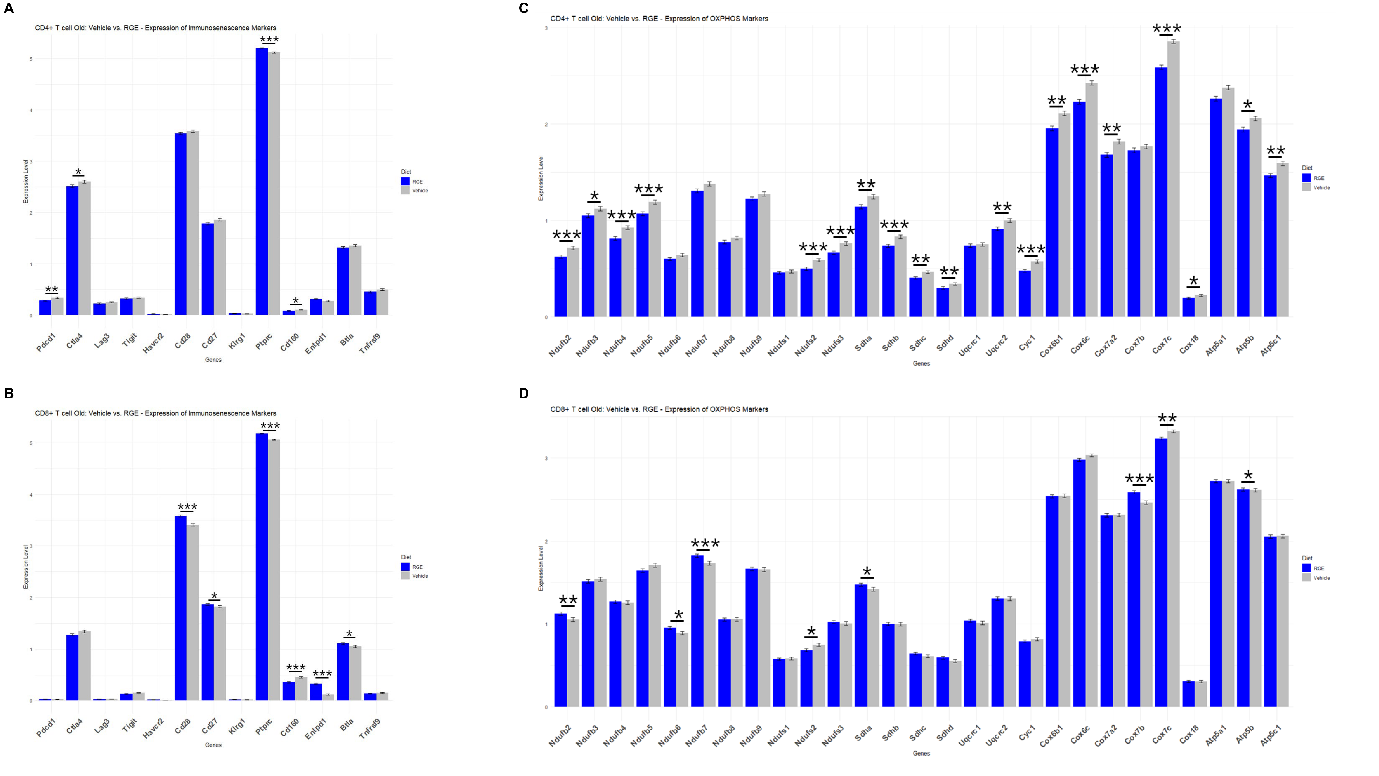


Supplemental Fig 6. Metabolic function in CD4+ and CD8+ T cells from RGE treated old mice. (A, B) Bar plot of T cell senescence marker in CD4+ T cell and CD8+ T cell using RGE treated young mice single cell transcriptomics. (C, D) Bar plot of T cell OXPHOS complex marker in CD4+ T cell and CD8+ T cell using RGE treated young mice single cell transcriptomics. Data are expressed as means ± standard deviation. *P < 0.05, **P < 0.01, and ***P < 0.001.
